# First Description of Intergenic Sequences in Corydoradinae and Introducing the Complete Mitogenome of *Hoplisoma concolor* (Siluriformes: Callichthyidae)

**DOI:** 10.3390/genes16030282

**Published:** 2025-02-26

**Authors:** Seong Duk Do, Jae-Sung Rhee

**Affiliations:** 1 Department of Marine Science, College of Natural Science, Incheon National University, Incheon 22012, Republic of Korea; 2 Research Institute of Basic Sciences, Incheon National University, Incheon 22012, Republic of Korea; 3 Yellow Sea Research Institute, Incheon 22012, Republic of Korea

**Keywords:** callichthyid, Callichthyinae, Corydoradinae, *Corydoras concolor*, phylogenetic analysis

## Abstract

**Background/Objectives**: In this study, we report the complete mitochondrial genome sequence of *Hoplisoma concolor* Weitzman, 1961 (Siluriformes: Callichthyidae), a callichthyid catfish. **Methods**: DNA sequencing was performed to obtain its complete mitogenome using the HiSeq platform. To assess the phylogenetic relationships, maximum-likelihood and Bayesian inference phylogenetic trees were constructed using two ribosomal RNA (rRNA) genes and all protein-coding sequences (PCGs) concatenated from the *H. concolor* mitogenome, along with 31 other Siluriformes mitogenomes. **Results**: The complete mitogenome of *H. concolor* is 16,579 base pairs in length, with a nucleotide composition of 32.2% A, 26.0% T, 15.3% G, and 26.5% C. It contains 13 PCGs, 22 transfer RNA genes, and 2 rRNA genes. Phylogenetic analysis based on all PCGs and two rRNAs of the complete mitogenome confirms *H. concolor* as a sister species of *H. panda* within the subfamily Corydoradinae. In addition, intergenic sequences between *atp6* and *cox3* of 21 species of Corydoradinae provide further support for their phylogenetic relationship. **Conclusions**: Given the lack of detailed descriptions regarding the length and nucleotide composition of these intergenic sequences, our study contributes valuable insights into the genetic diversity and evolutionary complexity of Callichthyidae.

## 1. Introduction

The order Siluriformes, commonly known as catfish, is one of the most diverse groups within Actinopterygii, comprising 40 families, 446 genera, and over 3000 species [1,2]. It accounts for approximately 10.8% of all fish species and 5.5% of all vertebrates [1]. They are widely distributed across the globe, with most members inhabiting freshwater environments, while two families, Ariidae and Plotosidae, have adapted to brackish or marine environments [2,3]. Siluriformes are economically important due to their role in aquaculture and popularity in the ornamental fish industry [2]. Morphologically, they share several common traits, such as small eyes, a lack of scales and, therefore, a naked body, and circumoral barbels around the mouth, which serve as mechanosensory and chemosensory organs. Siluriformes are typically bottom-dwelling, nocturnal, and well-adapted to their environments through unique feeding behaviors [2,4,5]. This order plays crucial roles in aquatic food webs, acting as predators, scavengers, and detritivores.

The family Callichthyidae, commonly known as armored catfish, belongs to the order Siluriformes and includes two subfamilies, Callichthyinae and Corydoradinae [6]. It comprises eight genera and over 200 species, primarily inhabiting neotropical ecosystems in South America [4,6]. Unlike most Siluriformes, callichthyid catfishes are relatively small and possess unique morphological and geographical traits, as they are covered with two distinctive rows of bony plates, have a blunt snout with an inferior mouth, and exhibit a dual breathing system that enables survival in low-oxygen environments [2,6,7].

The subfamily Corydoradinae has traditionally been classified based on phenotypic characteristics into three genera: *Corydoras*, the most species-rich genus with over 180 species, *Scleromystax*, and *Aspidoras* [8]. However, this classification remains controversial due to ambiguity arising from distinctive characteristics such as Müllerian mimicry, an evolutionary interaction between predator and prey [9]. DNA sequence-based classification is also debated within Corydoradinae. Molecular phylogenetic studies using partial mitogenomes and nuclear DNA have identified nine lineages within the subfamily, suggesting greater diversity than previously recognized [9]. Recent classification based on ultraconserved elements (UCEs) has redefined the taxonomy of Corydoradinae by expanding the number of recognized genera from three to seven: *Corydoras*, *Aspidoras*, *Scleromystax*, *Gastrodermus*, *Osteogaster*, *Brochis*, and *Hoplisoma* [10]. Therefore, additional genomic data should be accumulated to support phenotypic classifications and establish robust molecular evolutionary scenarios based on genetic divergence.

In this study, we aimed to contribute to resolving the complex molecular phylogenetic relationships within Corydoradinae by sequencing and reporting the complete mitochondrial genome of *H. concolor* Weitzman, 1961 (Siluriformes: Callichthyidae), as the mitogenome is considered a powerful tool for molecular phylogenetic classification. This species was previously classified under the genus *Corydoras* as *Corydoras concolor* but was recently reclassified into the genus *Hoplisoma* and renamed *H. concolor* [10]. Therefore, the mitogenome of *H. concolor*, presented here for the first time in the reclassified to the genus form, provides valuable data that will contribute to the molecular phylogenetic classification within Corydoradinae.

Another key contribution of our manuscript is highlighting the potential use of an intergenic sequence between the *atp6* and *cox3* genes within the Callichthyidae mitogenome as supportive evidence for molecular phylogeny. A synapomorphy feature of the Callichthyidae mitogenome is the presence of an *atp6*-*cox3* intergenic sequence. However, detailed information on these intergenic sequences and their relationships to phylogenetic distances among members of Corydoradinae remains limited. To address this gap, we analyzed the unique intergenic sequences within Corydoradinae and assessed their potential relationship with phylogenetic positions. Our findings, therefore, provide valuable insights into the genetic diversity and evolutionary complexity of Callichthyidae.

## 2. Materials and Methods

### 2.1. Fish and DNA Extraction

Individuals of *H. concolor* were obtained from the aquarium Magic Aqua (Incheon, Republic of Korea) (Figure S1). Muscle tissue from an individual was used for DNA extraction and cataloged at the Research Institute of Basic Sciences, Incheon National University, South Korea, under the Specimen ID 2024-Callichthyidae-07. Total genomic DNA was isolated using the DNeasy Blood and Tissue Kit (Qiagen, Hilden, Germany) following the manufacturer’s protocols.

### 2.2. DNA Sequencing, Assembly, and Gene Annotation

DNA sequencing was performed to obtain the complete mitogenome of *H. concolor* using the HiSeq platform (150 bp; HiSeq X ten; Illumina, San Diego, CA, USA) following our protocol [11]. Library preparation for Illumina HiSeq sequencing was constructed using the TruSeq DNA Sample Preparation Kit (Illumina) according to the manufacturer’s guidelines at Macrogen, Inc. (Seoul, Republic of Korea). This process involved the random fragmentation of purified DNA samples, followed by the ligation of 5′ and 3′ adapters. The prepared library was then sequenced on the Illumina HiSeq platform, and the paired-end raw reads underwent a stringent quality control process using FastQC version 0.11.9 [12]. After demultiplexing, only index-matched pairs were retained for further analysis. Raw read data were subjected to a rigorous quality trimming process using Trimmomatic [13], removing adapter sequences, low-quality reads, reads with more than 10% unknown bases, and those containing ambiguous bases. This process refined an initial total of 6,046,901,908 raw reads down to 40,045,708 high-quality filtered reads. To obtain an intact circular contig of the *H. concolor* mitogenome, a de novo assembly was performed using NOVOplasty [14] with various k-mer sizes. The resultant consensus sequence was annotated using MITOS2 [15], and a mitochondrial genome map was generated with Proksee [16].

### 2.3. Mitogenome Analysis

The nucleotide composition, codon frequency, and relative synonymous codon usage (RSCU) of the *H. concolor* mitogenome were analyzed using MEGA 11 version 11.0.3 [17]. AT-skew and GC-skew were calculated using the formulas AT-skew = (A − T)/(A + T) and GC-skew = (G − C)/(G + C), respectively. Secondary structure prediction of transfer RNA (tRNA) genes was conducted using the tRNAscan-SE Search Server [18]. The tandem repeat region within the control region was identified using Tandem Repeat Finder [19]. Intergenic sequences located between *atp6* and *cox3* genes in 21 species of Corydoradinae were retrieved from the NCBI database and aligned using MAFFT version 7.490 [20]. Uncorrected pairwise distance analysis for *atp6*-*cox3* intergenic sequences was conducted using MEGA 11 version 11.0.3 [17]. For the 13 PCGs of the 21 species of Corydoradinae listed in Table 1, K2P distances and nucleotide diversity (Pi) were calculated using MEGA 11 version 11.0.13 [17]. In addition, the Ka/Ks ratios were calculated using DnaSP version 6.12.03 [21].

### 2.4. Phylogenetic Analysis

To establish a robust molecular phylogenetic classification, we used complete sequences derived from 13 PCGs and two rRNA genes to construct the phylogenetic tree. To assess the phylogenetic relationships of *H. concolor*, maximum-likelihood (ML) and Bayesian inference (BI) phylogenetic trees were constructed using two ribosomal RNA (rRNA) genes and all protein-coding sequences (PCGs) concatenated from the *H. concolor* mitogenome, along with 31 other Siluriformes mitogenomes (21 Callichthyidae, 1 Trichomycteridae, 7 Loricariidae, and 2 Siluridae). The accession numbers of the sequences used in the phylogenetic analysis are provided in Table S1. The 12s and 16s rRNA genes, along with 13 PCGs from these species, were retrieved from the NCBI database.

Sequence alignment of the two rRNA genes and 13 PCGS was performed using the L-INS-I algorithms in MAFFT version 7.490 [20]. Redundant gaps were removed with trimAl v 1.4 [22], and the refined sequences were concatenated using SequenceMatrix version 1.8.1 and converted to Nexus form [23]. The optimal substitution model was determined using ModelFinder within IQ-TREE version 2.0.7 based on the Bayesian Information Criterion (BIC) (Table S2) [24]. The ML phylogenetic tree was constructed with 1000 ultrafast bootstrap replications in IQ-TREE2 version 2.0.7 [25].

For BI analysis, MrBayes version 3.2.7 was used with the best-fit substitution model selected based on the Akaike Information Criterion (AIC) [26]. Two independent MCMC runs of one million generations were performed with four chains, sampling every 100 generations. The effective sample size (ESS) was evaluated using Tracer version 1.7.2, with all metrics exceeding 200, except for the initial 25% burn-in [27]. A total of 20,002 trees were generated, with the first 25% discarded as burn-in using LogCombiner version 2.7, and a consensus tree was constructed using TreeAnnotator version 2.7.6 [28]. The final ML and BI phylogenetic trees were edited and visualized in Figtree version 1.4.4 [29].

## 3. Results

### 3.1. Mitogenome Structure

**Figure 1:**
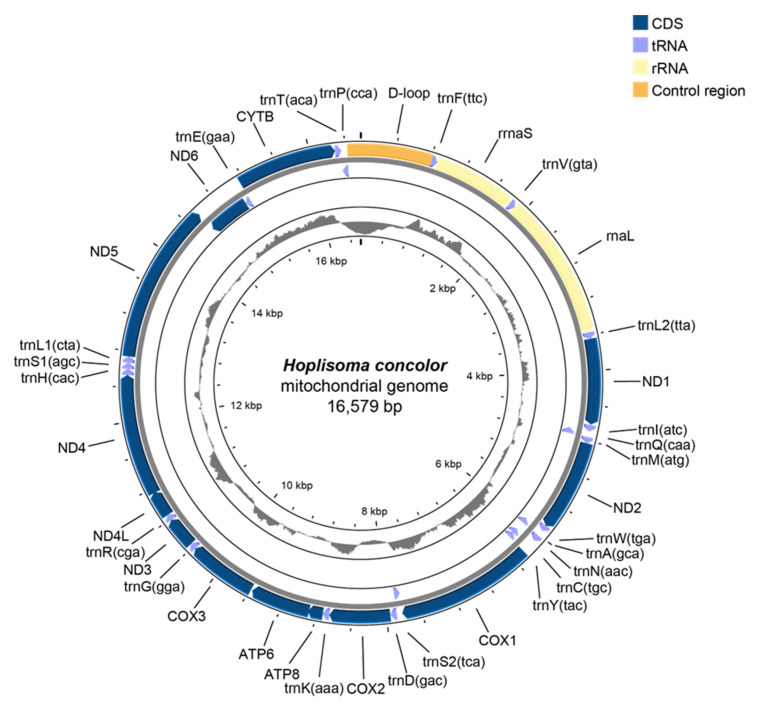
Circular map of the assembled *H. concolor* mitogenome, consisting of 13 PCGs, 22 tRNA, and 2 rRNA genes. Genes encoded on the reverse strand are illustrated inside the circle, while those on the forward strand are depicted outside the circles.

The complete mitogenome of *H. concolor* is 16,579 bp long (GenBank accession no. OQ569933). It contains a total of 13 PCGs, 2 rRNA genes such as 12S *rRNA* and 16S *rRNA*, and 22 tRNA genes (Figure 1, Table 1). Of these genes, 12 PCGs and both rRNA genes are encoded on the major strand, while only the *nad6* gene is encoded on the minor strand (Figure 1, Table 1). Among the 22 tRNAs, 14 (*trnD*, *trnF*, *trnG*, *trnH*, *trnI*, *trnK*, *trnL1*, *trnL2*, *trnM*, *trnR*, *trnS1*, *trnT*, *trnV*, and *trnW*) are located on the major stand, whereas the remaining 8 (*trnA*, *trnC*, *trnE*, *trnN*, *trnP*, *trnQ*, *trnS2*, and *trnY*) are on the minor strand (Figure 1, Table 1). The intergenic nucleotide region between *atp6* and *cox3* genes is 17 bp (Table 1). The control region (D-loop) is 966 bp long, with a 35 bp tandem repeat (Figure S2).

### 3.2. rRNA and tRNA Composition

**Figure 2:**
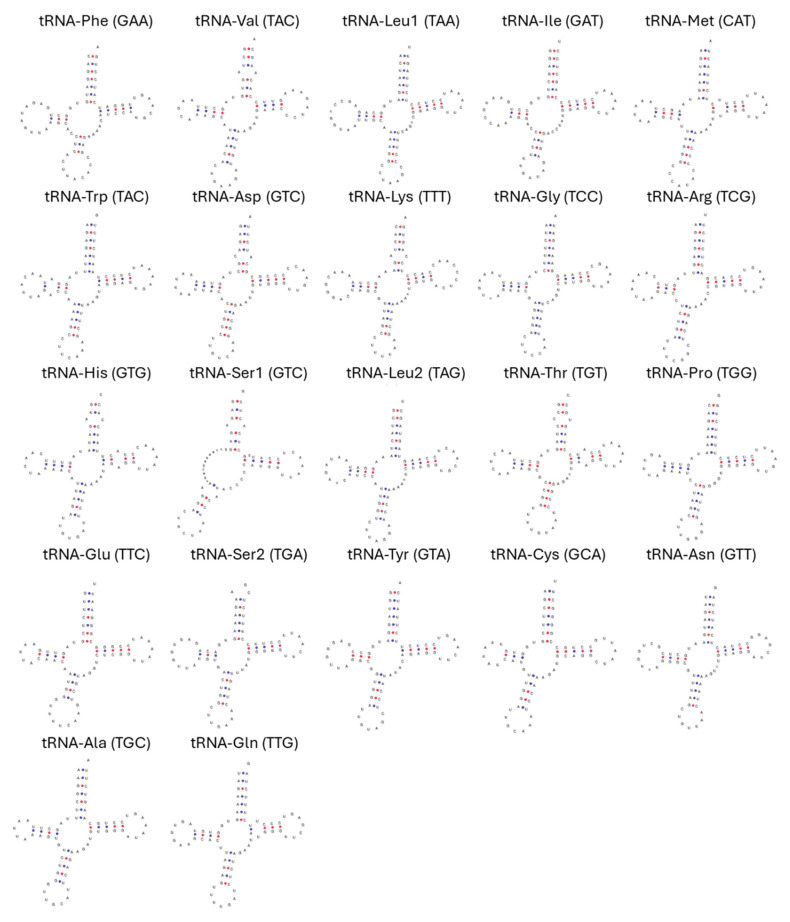
The predicted secondary structure of tRNAs in the *H. concolor* mitogenome includes information on the anticodon sequence of each tRNA and the corresponding amino acids they transport.

The two rRNA genes, 12S rRNA, and 16S rRNA, are 944 bp and 1670 bp long, respectively. The 12S rRNA gene is located between *trnF* and *trnV*, while the 16S rRNA gene is positioned between *trnV* and *trnL2* (Figure 2 and Table 1).

In the predicted secondary structure of the 22 tRNAs, including two copies of *trnS* and *trnL*, all tRNAs exhibited a typical cloverleaf structure except for *trnS1*, which had a deleted DHU arm (Figure 2). The total length of the tRNAs is 1561 bp, with individual tRNA lengths ranging from 58 to 75 bp. The shortest tRNA is *trnS1* (58 bp), while the longest is *trnL2* (75 bp) (Table 1).

### 3.3. Analysis of PCG Composition and Codon Usage

**Figure 3:**
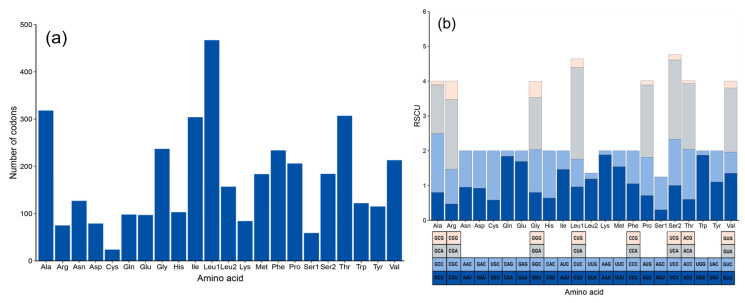
(**a**) Frequency of amino acid in the PCGs of the *H. concolor* mitogenome. (**b**) Relative synonymous codon usage (RSCU) in the PCGs of the *H. concolor* mitogenome.

Among the 13 PCGs, 12 initiate with the conventional start codon ATG, while the *cox1* gene begins with the start codon GTG. Six PCGs (*nad1*, *atp6*, *atp8*, *nad4l*, *nad5*, and *nad6*) terminate with the complete stop codons (TAA and TAG), whereas the *cox1* gene ends with AGG. In addition, *cox2*, *cox3*, *nad2*, *nad3*, *nad4*, and *cytb* conclude with an incomplete end codon (T-) (Table 1). The total length of the 13 PCGs is 11,412 bp, with *atp6* being the shortest (168 bp) and *nad5* the longest (1827 bp) (Table 1). The total number of amino acids in the PCGs is 3807, with the most frequently used amino acids being Leu1 (467, 12.27%), Ala (318, 8.35%), Thr (307, 8.06%), and Ile (304, 7.99%) (Figure 3a and Table S3). The least frequently used amino acids were Cys (24, 0.63%), Ser2 (59, 1.55%), Arg (74, 1.94%), and Asp (79, 2.08%) (Figure 3a and Table S3).

RSCU analysis of the 13 PCGs revealed that the most frequent codons were UCA (2.37, Ser), CUA (2.33, Leu1), CGA (2.32, Arg), and CCA (2.25, Pro), while the least frequent were GCG (0.1, Ala), UCG (0.1, Ser1), CGG (0.11, Arg), and CAG (0.12, Gln) (Figure 3b and Table S3). Among the 24 codons with an RSCU value of 1 or higher, 15 codons featured an A in the third position, indicating a preference for either A or T in the codon usage (Figure 3b and Table S3).

**Figure 4:**
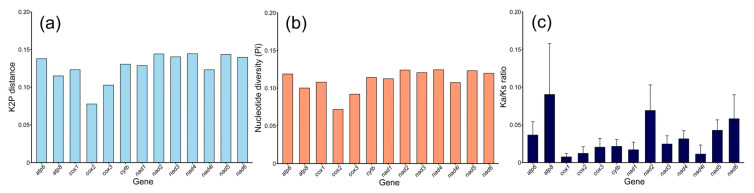
(**a**) K2P distance values, (**b**) nucleotide diversity (Pi) values, and (**c**) Ka/Ks ratio values for the 13 PCGs across 21 species of the Corydoradinae subfamily.

To determine the evolutionary relationships of the 13 PCGs across 21 species of Corydoradinae, analyses of K2P distance, Pi, and Ka/Ks ratios were conducted (Figure 4). The K2P distance analysis revealed that *cox2* had the lowest value (0.0777), followed by *cox3* (0.1027) and *atp8* (0.1149), while *nad4* had the highest value (0.1444), followed by *nad2* (0.1441) and *nad5* (0.1434) (Figure 4a). The results of the Pi analysis closely mirrored those of the K2P distance analysis, with *cox2* (0.0718), *cox3* (0.0920), and *atp8* (0.1001) exhibiting the lowest Pi values, whereas *nad4* (0.1244), *nad2* (0.1240), and *nad5* (0.1231) displayed the highest values (Figure 4b). The Ka/Ks ratios for all 13 PCGs were less than 1 (<1) (Figure 4c), indicating purifying selection. Among them, *cox1* had the lowest Ka/Ks ratio (0.00075), followed by *nad4l* (0.0113) and *cox2* (0.0122), whereas *atp8* exhibited the highest Ka/Ks ratio (0.0902), followed by *nad2* (0.0690) and *nad6* (0.0581) (Figure 4c).

### 3.4. Nucleotide Composition and Skewness

In the 16,579 bp mitogenome of *H. concolor*, the base composition is as follows: A, 32.2%; T, 26.01%; C, 26.49%; and G, 15.3%, resulting in an A + T content of 58.21% and a C + G content of 41.79%, indicating a predominance of AT base pairs (Table 2). The A + T contents of the different genes are as follows: PCGs, 57.69%; tRNA, 57.53%; rRNA, 57.42%; and the control region, 67.91%, with the control region exhibiting the highest AT bias (Table 2). Among the PCGs, the *atp8* gene had the highest A + T content (61.9%), while the *nad4l* gene had the lowest (50.84%) (Table 2).

The AT-skew values for the *H. concolor* mitogenome, as well as its PCGs, tRNAs, rRNAs, and control regions, were all positive, while the GC-skew values were generally negative, except for the tRNAs (Table 2). Among the 13 PCGs, the AT-skew values were positive for all genes except for *cox1*, *nad4l*, and *nad6*, with *nad2* showing the highest value and *nad6* the lowest, indicating a strong bias toward A and T bases, respectively. In contrast, the GC-skew values were negative for 12 PCGs, except for *nad6*, indicating a general bias toward C bases. Overall, the 13 PCGs exhibit a preference for A and C bases, with *nad6* being the only gene showing a bias toward T and G bases (Figure S3 and Table 2).

### 3.5. Phylogenetic Analysis

**Figure 5:**
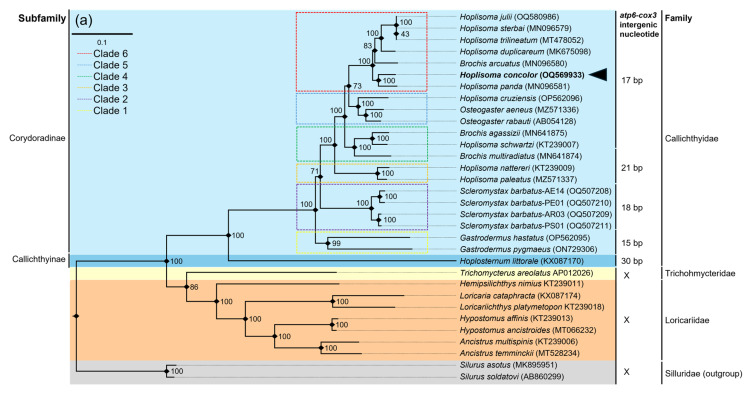

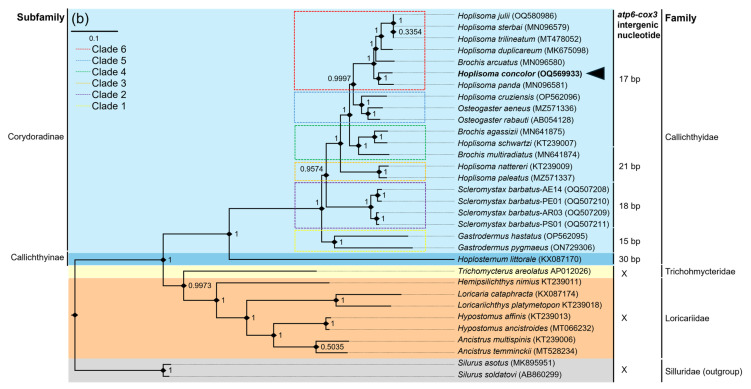
The phylogenetic tree of 32 published Siluriforme mitogenomes, including that of *H. concolor*, was constructed based on the concatenated nucleotide sequences of 13 PCGs and 2 rRNAs. (**a**) The numbers on the nodes indicate ML bootstrap percentages. (**b**) The numbers on the nodes indicate Bayesian posterior probability. Genbank accession numbers for the published sequences are incorporated. The base pair numbers indicated the number of intergenic nucleotides between *atp6* and *cox3* genes. Dashed boxes represent each clade. The black arrow represents the catfish analyzed in this study. References for the mitogenome data used in this analysis are appended in Table S1.

In a phylogenetic analysis using 13 PCGs and 2 rRNAs from 32 siluriform species, the ML and BI trees exhibited identical topologies (Figure 5a,b). Both the ML and BI trees strongly supported the monophyly of Callichthyidae, with high bootstrap and posterior probability values. Additionally, the subfamilies Callichthyinae and Corydoradinae were confirmed as monophyletic groups with strong statistical support (BS: 100, PP: 1). The genera *Gastrodemus* and *Scleromystax* formed a single clade, while *Hoplisoma*, *Brochis*, and *Osteogaster* did not constitute a monophyletic group.

*H. concolor* was positioned as the sister species to *H. panda* with strong statistical support (BS: 100, PP: 1) (Figure 5a,b). The ML and BI trees constructed using only 13 PCGs exhibited the same topologies as those based on 13 PCGs and 2 rRNA genes, with *H. concolor* forming a sister species relationship with *H. panda*, with high support values (BS:100, PP: 1) (Figures S4 and S5).

The 30 bp *atp6*-*cox3* intergenic sequence observed in *Hoplosternum littorale*, a species of the Callichthyinae, is generally longer than the 15–21 bp typically observed in Corydoradinae species (Figure 5a,b). A correlation is observed between *atp6*-*cox3* intergenic sequence length and clade placement in the phylogenetic tree. For example, clade 1 (*Gastrodermus hastatus* + *G. pygmaeus*) has a 15 bp intergenic sequence, while clade 2, represented by *S. barbatus*, a member of the genus *Scleromystax*, has an 18 bp intergenic sequence of 18 bp. In the phylogenetic tree, clade 3, consisting of *H. nattereri* and *H. paleatus*, has a 21 bp intergenic sequence and forms a monophyletic group. In addition, clades 5 and 6 share a 17 bp intergenic sequence across all species.

**Figure 6:**
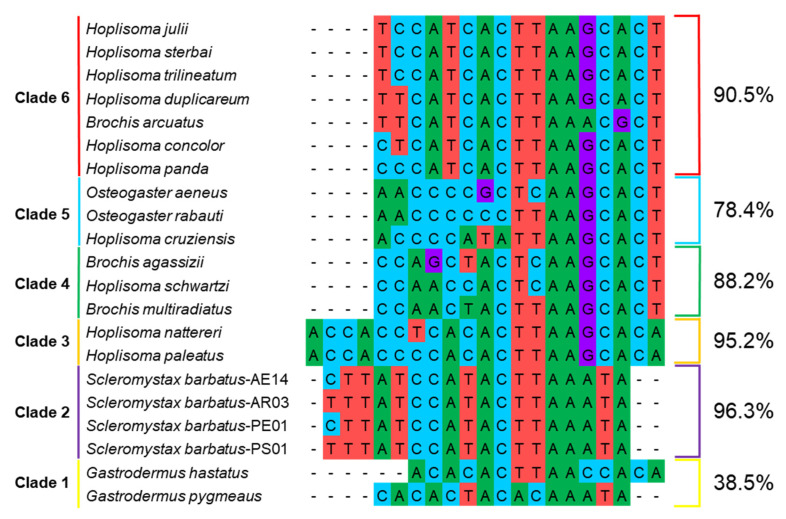
The intergenic sequences between *atp6* and *cox3* for 21 species within Corydoradinae. The percentages on the right represent the average sequence identity values, calculated based on uncorrected pairwise distance (p-distance) between species within the same clade. The p-distance values for all species are provided in Table S4.

Clade 1, consisting of *G. hastatus + G. pygmaeus*, had an *atp6*-*cox3* intergenic region characterized by (A)CACACTWMACMAMW(A) (Figure 6). Clade 2, represented by *S. barbatus*, contained the sequence YTTATCCATACTTAAATA. Clade 3, consisting of *H. nattereri* and *H. paleatus*, had an *atp6*-*cox3* intergenic sequence of ACCACCYCACACTTAAGCACA. Clades 4 and 5 included [(*Brochis agassizii + H. schwartzi*) + *B. multiradiatus*] and [(*Osteogaster aeneus + O. rabauti*) *+ H. cruziensis*], respectively, which exhibited the sequences CCARCYACTYAAGCACT and AMCCCMBMTYAAGCACT. Lastly, clade 6, comprising [(((*H. sterbai + H. trilineatum*) + *H. duplicareum*) + *H. panda*) + (*H. concolor + B. arcuatus*)], was characterized by the intergenic region YYCATCACTTAARCRCT. In the comparison of *atp6*-*cox3* intergenic sequences between clades based on uncorrected pairwise distance, the highest identity percentage was found in the same clade, except for clade 1 (Figure S6 and Table S4). In addition, identity percentages were generally higher within clades than between clades.

## 4. Discussion

This study provides the complete mitochondrial genome of *H. concolor*, a member of the subfamily Corydoradinae. Following the reclassification of genera within Corydoradinae, this is the first to utilize complete mitogenomes from the newly classified genera. Although the total length of the *H. concolor* mitogenome was relatively shorter than those of other Corydoradinae species sequenced to date, ranging from 16,518 to 16,916 bp, its nucleotide and amino acid composition showed no significant differences from other species within the subfamily. The composition and arrangement of 13 PCGs, 2 rRNAs, and 22 tRNAs genes observed in the mitogenome of *H. concolor* were consistent with those found in general Corydoradinae, which are also identical to those observed in vertebrate mitochondria [1,2,3,4,5,6,7,8,9,10,11]. The start codons for the 13 PCGs in *H. concolor* were ATG, except for *cox1*; and *cox3*, *nad2*, *nad3*, *nad4*, and *cytb* ended with an incomplete termination codon. This is a common feature in fish mitogenomes, where incomplete termination codons are known to function as normal stop codons through post-transcriptional polyadenylation [12].

A synonymous codon is a codon that encodes the same amino acid but is used disproportionately due to selective pressure from the environment [13]. Analyzing synonymous codon usage can provide insights into the evolutionary dynamics of a species’ mitogenome. In the RSCU analysis conducted in this study, a strong anti-G bias was observed at the third codon position. An RSCU value of 1.0 indicates equal usage of a codon, while values above 1.0 suggest more frequent usage than expected, and values below 1.0 indicate less frequent usage. This phenomenon is common in metazoans and may be influenced by factors such as high AT content or the proportion of tRNA in the mitochondria [14]. In addition, the nucleotide and amino acid composition of the *H. concolor* mitogenome, along with the deletion of the DHU arm observed in the predicted secondary structure of *trnS1*, are consistent with previous studies on the mitogenomes of Corydoradinae [1,8].

The phylogenetic tree recovered in this study is highly consistent with previous studies using either 13 PCGs alone or 13 PCGs combined with 2 rRNAs [1,8,9]. However, the topology of the ML/BI tree constructed using two rRNAs, *cytb*, *nd4*, and *trna* from 452 species within Callichthyidae revealed a total of nine lineages in Corydoradinae [15]. Recently, an ML tree on 156 UCEs from Callichthyidae identified seven lineages. Based on these molecular results and morphological characteristics, Corydoradinae, which was previously composed of three genera, has been reorganized into seven genera: *Corydoras* (lineage 1), *Aspidoras* (lineage 2), *Scleromystax* (lineage 3), *Gastrodermus* (lineages 4 and 5), *Osteogaster* (lineage 7), *Brochis* (lineage 8), and *Hoplisoma* (lineages 6 and 9) [16].

The majority of species (>90%) within the family Callichthyidae belong to the subfamily Corydoradinae, yet complete mitochondrial genomes remain unavailable for many of these species [17]. Consequently, if only a limited number of species were used in constructing the phylogenetic tree, this may not fully explain why species within the genera *Brochis*, *Osteogaster*, and *Hoplisoma* fail to form a monophyletic clade. Additionally, the inclusion of only *S. barbatus* in the phylogenetic analysis, without representatives from the genera *Corydoras* and *Aspidoras* (lineages 1 and 2), may account for the discrepancy between the previous genus classification—[((*Brochis* + *Osteogaster*) + *Hoplisoma*) + (*Gastrodermus*) + (*Scleromystax + Aspidoras*) + (*Corydoras*)]—and the results of the present study. Therefore, further studies on the mitogenomes of species within the Corydoradinae subfamily, including the genera *Corydoras*, *Aspidoras*, and *Scleromystax*, are necessary to clarify the cryptic phylogenetic relationships within Corydoradinae.

The results of the ML/BI phylogenetic trees using 13 PCGs with 2 rRNAs, as well as 13 PCGs alone, suggest the existence of six clades within Corydoradinae. This finding closely aligns with the multiple sequence alignment analysis of the *atp6*-*cox3* intergenic sequences. Although the presence of an intergenic sequence between the *atp6* and *cox3* genes is a unique feature of the Callichthyidae mitogenome, there is a lack of detailed explanations regarding its variations across genera and species, as well as its potential contribution to understanding phylogenetic relationships. Despite the maternal inheritance and high conservation of the mitogenome, *atp6*-*cox3* intergenic sequences vary between clades as briefly observed in Callichthyidae [18,19].

Overall, the similarity of *atp6*-*cox3* intergenic sequences within a clade indicates that these sequences tend to be more conserved, especially among sister species. In clade 6, which had an *atp6*-*cox3* intergenic sequence of YYCATCACTTAARCRCT, the sequences of *H. julii*, *H. sterbai*, and *H. trilineatum* were perfectly matched, all sharing the sequence TCCATCACTTAAGCACT. Among them, *H. julii* and *H. trilineatum* are morphologically very similar and are often confused [5]. In addition, previous studies have emphasized the genetic similarity between *H. sterbai* and *H. trilineatum* [8,30,31], and our results further support the close genetic relationship among *H. julii*, *H. sterbai*, and *H. trilineatum*. Moreover, within clade 6, the sister species *H. concolor* and *H. panda* exhibited the same sequence CYCATCACTTAAGCACT. In clade 5, *O. aeneus* and *O. rabauti* shared the sequence AACCCCSCTYAAGCACT, while in clade 4, *B. agassizii* and *H. schwartzi* exhibited CCARCYACTCAAGCACT. Clade 3 displayed the sequence ACCACCYCACACTTAAGCACA, with only one or two nucleotide substitutions observed between species within each clade. The four members of clade 2, despite consisting of only a single species, *S. barbatus*, exhibited habitat-related differences yet shared the *atp6*-*cox3* intergenic sequence YTTATCCATACTTAAATA. This pattern was consistent with the phylogenetic relationships within *S. barbatus* as indicated by the phylogenetic tree [20]. In addition, the use of only *S. barbatus* specimens from different geographic regions in clade 2 is a limitation of the clade 2 comparison.

The relatively higher similarity among members within a clade and the noticeable differences between clades 2–6 (78.4–96.3%) suggest that analyzing the *atp6*-*cox3* intergenic sequences may be a useful approach for phylogenetic classification in the complex members of Corydoradinae. Nevertheless, in clade 1, *O. hastatus* and *O. pygmaeus* exhibited a high p-distance for the atp6-cox3 intergenic sequences, along with long branch lengths in both the ML and BI phylogenetic trees. This pattern is likely the result of a high rate of nucleotide substitutions. However, further studies on species within the genera *Gastrodermus*, *Osteogaster*, and *Scleromystax*, in addition to their complete mitogenome data, are needed to clarify discrepancies and address the complexity of their molecular phylogeny, thereby improving our understanding of the relationships within this clade.

In conclusion, despite the intricate phylogenetic relationships within Corydoradinae, our findings suggest that phylogenetic classification based on traditional complete mitogenome sequences, combined with analyses of the *atp6*-*cox3* intergenic sequence and synapomorphy features of Callichthyidae, provides valuable evidence for understanding the complex phylogeny of Corydoradinae. In particular, we assume that these insights will contribute significantly to refining the phylogeny of the newly classified Corydoradinae.

## Figures and Tables

**Table 1:** Detailed information about the *H. concolor* mitogenome includes the start and end positions of each gene, the number of overlapping and *atp6*-*cox3* intergenic nucleotides, and the strand positions of genes, as well as the start and stop codons of entire PCGs and the anticodons of tRNAs.

Gene	Position					Codon		
	*H. concolor*				Strand	Start	Stop	Anticodon
	Start	End	Length (bp)	Intergenic Nucleotide				
*trnF*	1	68	68	0	H			GAA
*rrnS*	69	1012	944	0	H			
*trnV*	1013	1084	72	0	H			TAC
*rrnL*	1085	2754	1670	−1	H			
*trnL2*	2754	2828	75	0	H			TAA
*nad1*	2829	3800	972	7	H	ATG	TAA	
*trnI*	3808	3880	73	−2	H			GAT
*trnQ*	3879	3949	71	−1	L			TTG
*trnM*	3949	4018	70	0	H			CAT
*nad2*	4019	5063	1045	0	H	ATG	T-	
*trnW*	5064	5134	71	1	H			TCA
*trnA*	5136	5204	69	0	L			TGC
*trnN*	5205	5278	74	31	L			GTT
*trnC*	5310	5377	68	−1	L			GCA
*trnY*	5377	5446	70	1	L			GTA
*cox1*	5448	7007	1560	−13	H	GTG	AGG	
*trnS2*	6995	7065	71	4	L			TGA
*trnD*	7070	7140	71	3	H			GTC
*cox2*	7144	7834	691	0	H	ATG	T-	
*trnK*	7835	7908	74	1	H			TTT
*atp8*	7910	8077	168	−10	H	ATG	TAA	
*atp6*	8068	8751	684	17	H	ATG	TAG	
*cox3*	8769	9552	784	0	H	ATG	T-	
*trnG*	9553	9624	72	0	H			TCC
*nad3*	9625	9973	349	0	H	ATG	T-	
*trnR*	9974	10,043	70	0	H			TCG
*nad4l*	10,044	10,340	297	−7	H	ATG	TAA	
*nad4*	10,334	11,714	1381	0	H	ATG	T-	
*trnH*	11,715	11,784	70	0	H			GTG
*trnS1*	11,785	11,842	58	10	H			GCT
*trnL1*	11,853	11,925	73	0	H			TAG
*nad5*	11,926	13,752	1827	−4	H	ATG	TAA	
*nad6*	13,749	14,264	516	0	L	ATG	TAA	
*trnE*	14,265	14,333	69	2	L			TTC
*Cytb*	14,336	15,473	1138	0	H	ATG	T-	
*trnT*	15,474	15,545	72	−2	H			TGT
*trnP*	15,544	15,613	70	0	L			TGG
*D-loop*	15,614	16,579	966	0				

**Table 2:** Nucleotide composition and base-pair skewness on the *H. concolor* mitogenome.

Region	Size (bp)	A (%)	T (%)	C (%)	G (%)	A + T (%)	C + G (%)	AT-Skew	GC-Skew
Mitogenome	16,579	32.2	26.01	26.49	15.3	58.21	41.79	0.11	−0.27
*nad1*	972	30.56	27.16	28.6	13.68	57.72	42.28	0.06	−0.35
*nad2*	1045	35.12	24.21	29.76	10.91	59.33	40.67	0.18	−0.46
*cox1*	1560	26.86	28.65	26.67	17.82	55.51	44.49	−0.03	−0.2
*cox2*	691	31.55	27.06	25.76	15.63	58.61	41.39	0.08	−0.24
*atp8*	168	35.71	26.19	26.79	11.31	61.9	38.1	0.15	−0.41
*atp6*	684	31.14	28.36	27.05	13.45	59.5	40.5	0.05	−0.34
*cox3*	784	28.44	27.81	27.42	16.33	56.25	43.75	0.01	−0.25
*nad3*	349	30.09	30.09	26.93	12.89	60.17	39.83	0	−0.35
*nad4l*	297	22.56	28.28	31.99	17.17	50.84	49.16	−0.11	−0.3
*nad4*	1381	31.5	27.59	27.15	13.76	59.09	40.91	0.07	−0.33
*nad5*	1827	32.57	26.71	28.19	12.53	59.28	40.72	0.1	−0.38
*nad6*	516	14.76	41.55	11.26	32.43	56.31	43.69	−0.48	0.48
*Cytb*	1138	28.3	26.98	30.58	14.15	55.27	44.73	0.02	−0.37
PCGs	11,412	29.77	27.92	27.28	15.03	57.69	42.31	0.03	−0.29
tRNAs	1561	30.05	27.48	20.24	22.23	57.53	42.47	0.04	0.05
rRNAs	2614	35.88	21.54	22.95	19.63	57.42	42.58	0.25	−0.08
C.R.	966	36.75	31.16	18.74	13.35	67.91	32.09	0.08	−0.17

## Data Availability

BioProject, BioSample, and SRA accession numbers are PRJNA941372, SAMN33603757, and SRR23730154, respectively. The data that support the findings of this study are openly available in the National Center for Biotechnology Information with an accession number OQ569933.

## Supplementary Materials

The following supporting information can be downloaded at: https://www.mdpi.com/article/10.3390/genes16030282/s1, References [31,32,33,34,35,36,37,38,39,40,41,42,43,44,45,46,47] are cited in the Supplementary Materials. Figure S1: Photograph of *H. concolor* used for analysis; Figure S2: Tandem repeat sequence in control region of the *H. concolor* mitogenome; Figure S3: AT and GC-skewness of the 13 PCGs in the *H. concolor* mitogenome; Figure S4: A maximum likelihood (ML) phylogenetic tree of 32 published mitogenomes of Siluriformes, including that of *H. concolor*, based on the concatenated nucleotide sequences of 13 PCGs; Figure S5: A Bayesian phylogenetic tree of 32 published mitogenomes of Siluriformes, including that of *H. concolor*, based on the concatenated nucleotide sequences of 13 PCGs; Figure S6: The number of lengths for the uncorrected p-distance of the *atp6*-*cox3* intergenic sequence between and within clades is presented for 21 species belonging to the family Corydoradinae; Table S1: The mitogenome data used in phylogenetic analysis includes information; Table S2: Best substitution model used for phylogenetic trees; Table S3: Details on codon count and RSCU in the *H. concolor* mitogenome; Table S4: Uncorrected pairwise distance (p-distance) values among 21 species in the family Corydoradinae.
