# Supplementary material for: First Description of Intergenic Sequences in Corydoradinae and Introducing the Complete Mitogenome of Hoplisoma concolor (Siluriformes: Callichthyidae)
Source: Genes (Basel). 2025 Feb 26;16(3):282. doi: 10.3390/genes16030282 (PMC11942276; doi:10.3390/genes16030282)
Supplement: Supplementary file 1 [file genes-16-00282-s001.zip › genes-3492756-supplementary.pdf]

< Supplementary Information >

First description on intergenic sequences in Corydoradinae with introducing the complete mitogenome of *Hoplisoma concolor* (Siluriformes: Callichthyidae)

Seong Duk Do[a], Jae-Sung Rhee[a,b,c,*]

[a]Department of Marine Science, College of Natural Sciences, Incheon National University, Incheon, South Korea; [b]Research Institute of Basic Sciences, Incheon National University, Incheon, South Korea; [c]Yellow Sea Research Institute, Incheon, South Korea

*Correspondence:
jsrhee@inu.ac.kr (J.-S. Rhee)

TABLE OF CONTENTS

SUPPORTING INFORMATION......3
Figure S1......3
Figure S2.......4
Figure S3.......5
Figure S4.......6
Figure S5.......7
Figure S6.......8
Table S1.......9
Table S2.......11
Table S3.......12
Table S4.......13
Reference......Error! Bookmark not defined.

SUPPORTING INFORMATION

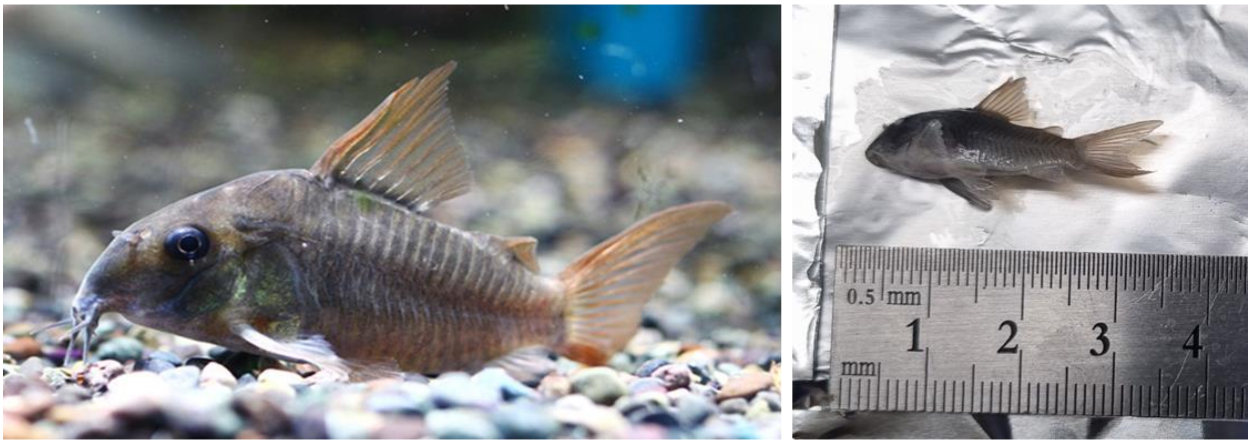

Figure S1. Photograph for *H. concolor* analyzed in this study. The photo was taken by fish specialist Woo Sang Cho (Magic Aqua, Incheon South Korea) and is kindly provided with permission.

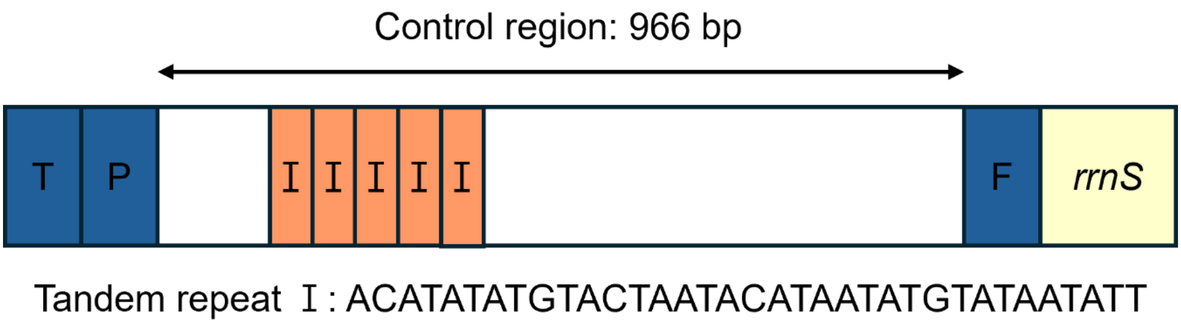

Figure S2. Tandem repeat sequence in control region of the *H. concolor* mitogenome. The 35 bp repeat sequence observed within the control region is highlighted with ‘I’.

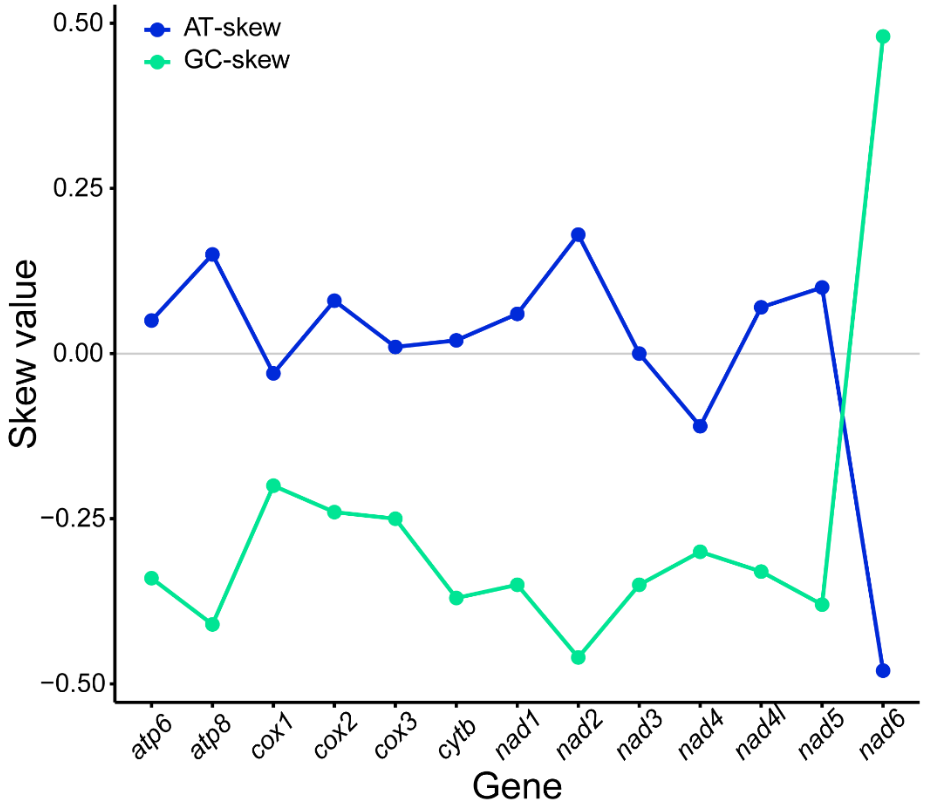

Figure S3. AT and GC-skewness of the 13 PCGs in the *H. concolor* mitogenome.

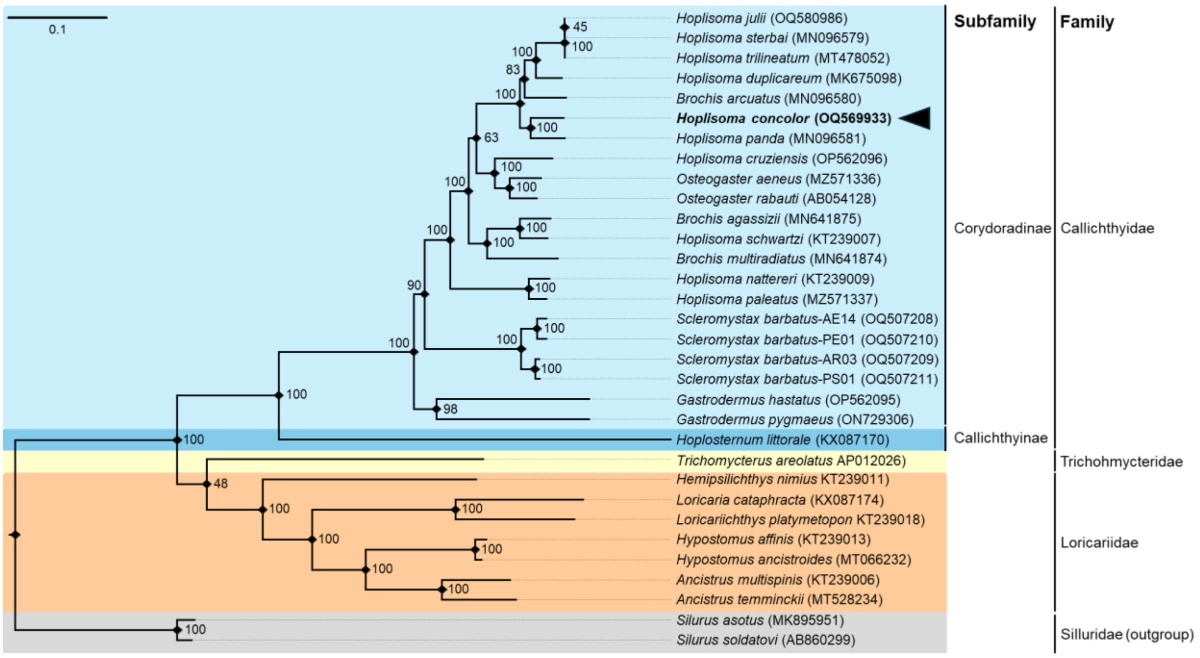

Figure S4. The phylogenetic tree of 32 published mitogenomes of siluriformes, including that of H. concolor, based on the concatenated nucleotide sequences of 13 PCGs. The numbers on the nodes indicate ML bootstrap percentages. Genbank accession numbers for published sequences are incorporated. The black arrow represents the catfish analyzed in this study. Reference on the mitogenome data used in this analysis is appended in **Table 1**.

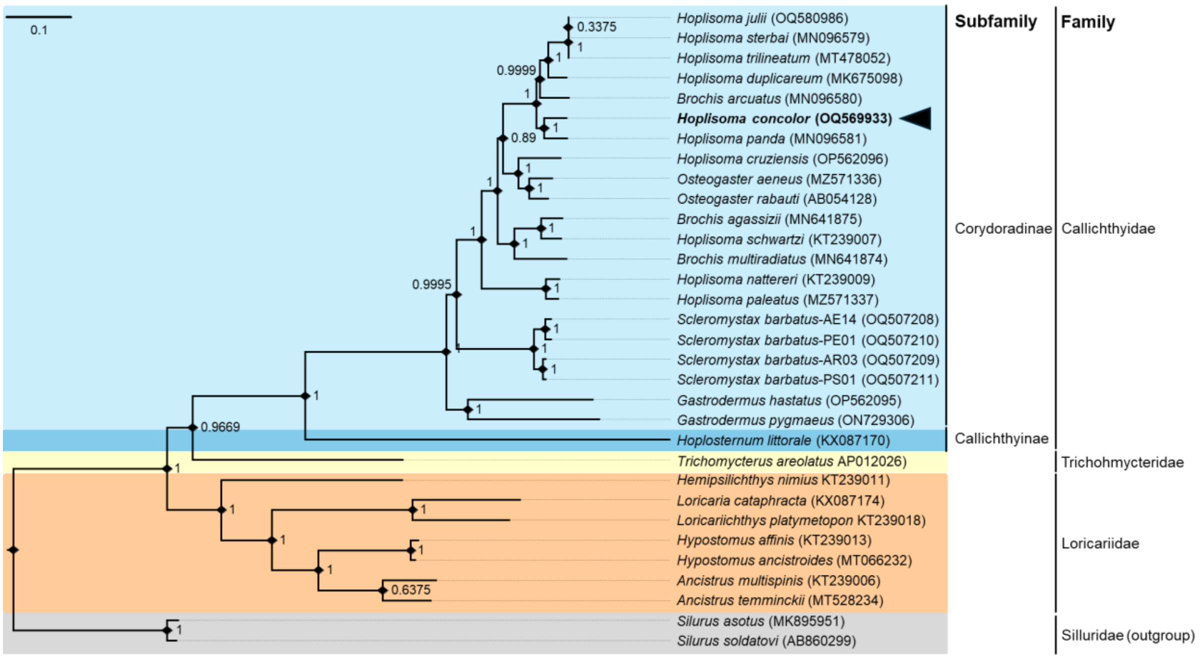

Figure S5. The phylogenetic tree of 32 published mitogenomes of siluriformes, including that of H. concolor, based on the concatenated nucleotide sequences of 13 PCGs. The numbers on the nodes indicate Bayesian posterior probability. Genbank accession numbers for published sequences are incorporated. The black arrow represents the catfish analyzed in this study. Reference on the mitogenome data used in this analysis is appended in **Table 1**.

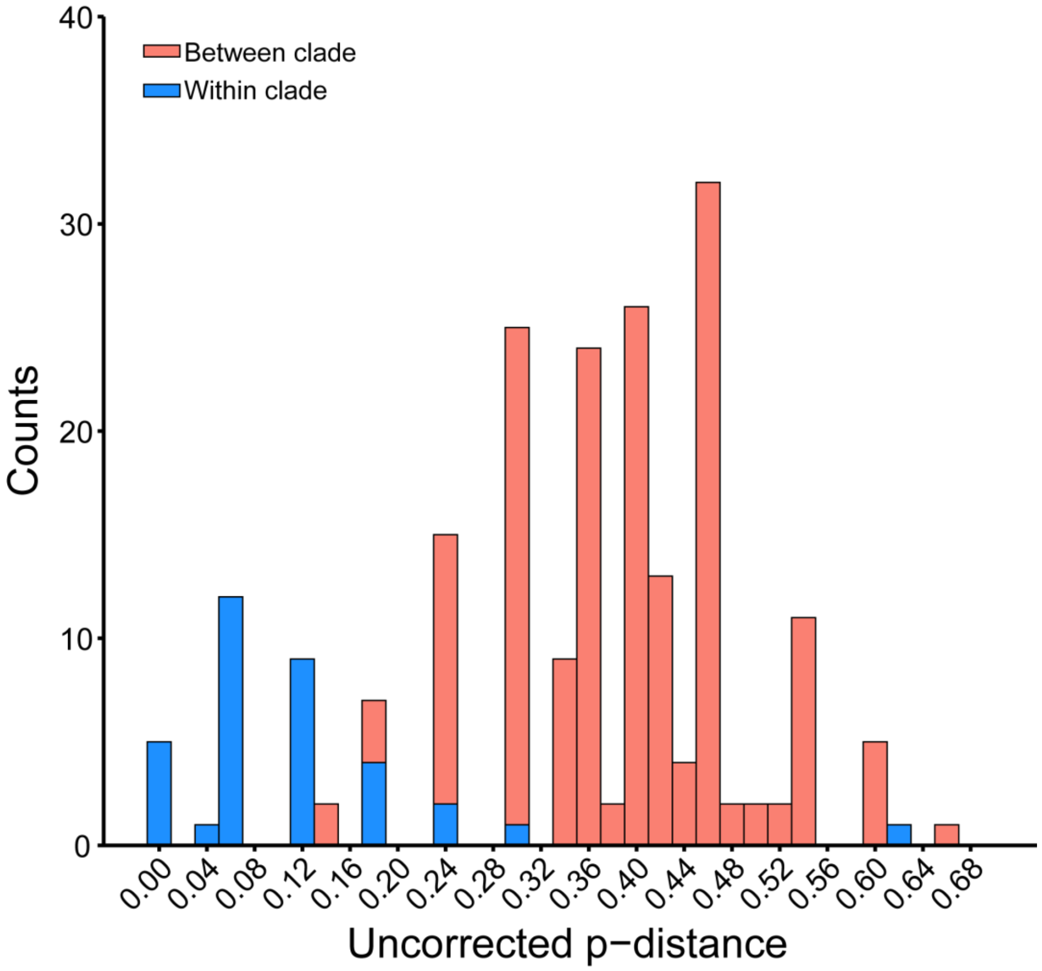

Figure S6. The number of lengths for the uncorrected p-distance of the atp6-cox3 intergenic sequence between and within clades is presented for 21 species belonging to the family Corydoradinae, with blue bars indicating within clades and orange bars indicating between clades.

Table S1. The mitogenome data used in the phylogenetic analysis includes information on the species, genus, and family, mitogenome length, NCBI accession number, and reference for each organism.

Family	Genus	Species	Length (bp)	Accession number	Reference
Callichthyidae	*Hoplisoma*	*Hoplisoma concolor*	16,579	OQ569933	This study
	Hoplisoma	*Hoplisoma duplicareum*	16,667	MK675098	[32]
	Hoplisoma	*Hoplisoma sterbai*	16,636	MN096579	[33]
	Hoplisoma	*Hoplisoma trilineatum*	16,526	MT478052	[34]
	Hoplisoma	*Hoplisoma panda*	16,611	MN096581	[35]
	Hoplisoma	*Hoplisoma julii*	16,636	OQ850956	Unpublished
	Hoplisoma	*Hoplisoma cruziensis*	16,531	OP562096	[31]
	Hoplisoma	*Hoplisoma schwartzi*	16,632	KT239007	[36]
	Hoplisoma	*Hoplisoma nattereri*	16,557	KT239009	[37]
	Hoplisoma	*Hoplisoma paleatus*	16,593	MZ571337	[30]
	Osteogaster	*Osteogaster rabauti*	16,831	AB054128	[38]
	Osteogaster	*Osteogaster aeneus*	16,604	MZ571336	[30]
	Brochis	*Brochis arcuatus*	16,822	MN096580	[39]
	Brochis	*Brochis agassizii*	16,562	MN641875	[40]
	Brochis	*Brochis multiradiatus*	16,916	MN641874	[41]
	Gastrodemus	*Gastrodemus hastatus*	16,518	OP562095	[31]
	Gastrodemus	*Gastrodemus pygmaeus*	16,840	ON729306	[31]
	Scleromystax	*Scleromystax barbatus*	16,651	OQ507208	[42]
	Scleromystax	*Scleromystax barbatus*	16,614	OQ507210	[42]
	Scleromystax	*Scleromystax barbatus*	16,694	OQ507209	[42]
	Scleromystax	*Scleromystax barbatus*	16,693	OQ507211	[42]
	Hoplosternum	*Hoplosternum littorale*	16,597 (P)	KX087170	[43]
Trichomycteridae	*Trichomycterus*	*Trichomycterus areolatus*	16,586 (P)	AP012026	[43]
Loricariidae	*Hemipsilichthys*	*Hemipsilichthys nimius*	16,320 (P)	KT239011	[37]
	Loricariichthys	*Loricariichthys platymetopon*	16,262 (P)	KT239018	[37]
	Loricaria	*Loricaria cataphracta*	16,409 (P)	KX087174	[37]
	Hypostomus	*Hypostomus affinis*	16,398 (P)	KT239013	[37]
	Hypostomus	*Hypostomus ancistroides*	16,826 (P)	MT066232	[44]

	Ancistrus	*Ancistrus multispinis*	16,177 (P)	KT239006	[37]
	Ancistrus	*Ancistrus temminckii*	16,557	MT528234	[45]
Siluridae	*Silurus*	*Silurus asotus*	17,385	MK895951	[46]
	Silurus	*Silurus soldatovi*	17,385	AB860299	[47]

Note: Patial mitogenomes were indicated with ‘P’.

Table S2. Best substitution model used for phylogenetic trees.

Gene	P123/P123+2rRNA (ML, BIC)	P123/P123+2rRNA (BI, AIC)
atp6	GTR+F+I+G4/GTR+F+I+G4	GTR+F+I+G4/GTR+F+I+G4
atp8	GTR+F+I+G4/GTR+F+I+G4	GTR+F+I+G4/HKY+F+I+G4
cox1	GTR+F+I+G4/GTR+F+I+G4	GTR+F+I+G4/GTR+F+I+G4
cox2	GTR+F+I+G4/GTR+F+I+G4	GTR+F+I+G4/GTR+F+I+G4
cox3	GTR+F+I+G4/GTR+F+I+G4	GTR+F+I+G4/GTR+F+I+G4
cytb	GTR+F+I+G4/GTR+F+I+G4	GTR+F+I+G4/GTR+F+I+G4
nad1	GTR+F+I+G4/GTR+F+I+G4	GTR+F+I+G4/GTR+F+I+G4
nad2	GTR+F+I+G4/GTR+F+I+G4	GTR+F+I+G4/GTR+F+I+G4
nad3	GTR+F+I+G4/GTR+F+I+G4	GTR+F+I+G4/GTR+F+I+G4
nad4	GTR+F+I+G4/GTR+F+I+G4	GTR+F+I+G4/GTR+F+I+G4
nad4l	GTR+F+I+G4/GTR+F+I+G4	GTR+F+I+G4/GTR+F+I+G4
nad5	GTR+F+I+G4/GTR+F+I+G4	GTR+F+I+G4/GTR+F+I+G4
nad6	HKY+F+I+G4/HKY+F+I+G4	HKY+F+I+G4/HKY+F+I+G4
rrnS	-/TIM2+F+I+G4	-/GTR+F+I+G4
rrnL	-/TIM2+F+I+G4	-/GTR+F+I+G4

Table S3. Details on codon count and RSCU in the *H. concolor* mitogenome.

Codon	Count	RSCU	Codon	Count	RSCU	Codon	Count	RSCU	Codon	Count	RSCU
UUU(F)	117	1	UCU(S)	30	0.74	UAU(Y)	45	0.78	UGU(C)	12	1
UUC(F)	117	1	UCC(S)	54	1.33	UAC(Y)	70	1.22	UGC(C)	12	1
UUA(L)	132	1.27	UCA(S)	96	2.37	UAA(*)	11	3.38	UGA(W)	112	1.84
UUG(L)	25	0.24	UCG(S)	4	0.1	UAG(*)	1	0.31	UGG(W)	10	0.16
CUU(L)	92	0.88	CCU(P)	26	0.5	CAU(H)	26	0.5	CGU(R)	11	0.59
CUC(L)	96	0.92	CCC(P)	49	0.95	CAC(H)	77	1.5	CGC(R)	18	0.97
CUA(L)	242	2.33	CCA(P)	116	2.25	CAA(Q)	92	1.88	CGA(R)	43	2.32
CUG(L)	37	0.36	CCG(P)	15	0.29	CAG(Q)	6	0.12	CGG(R)	2	0.11
AUU(I)	177	1.16	ACU(T)	28	0.36	AAU(N)	46	0.72	AGU(S)	11	0.27
AUC(I)	127	0.84	ACC(T)	109	1.42	AAC(N)	81	1.28	AGC(S)	48	1.19
AUA(M)	128	1.38	ACA(T)	158	2.06	AAA(K)	78	1.86	AGA(*)	0	0
AUG(M)	57	0.62	ACG(T)	12	0.16	AAG(K)	6	0.14	AGG(*)	1	0.31
GUU(V)	47	0.88	GCU(A)	52	0.65	GAU(D)	26	0.66	GGU(G)	31	0.52
GUC(V)	39	0.73	GCC(A)	128	1.61	GAC(D)	53	1.34	GGC(G)	71	1.2
GUA(V)	107	2.01	GCA(A)	130	1.64	GAA(E)	78	1.61	GGA(G)	104	1.76
GUG(V)	20	0.38	GCG(A)	8	0.1	GAG(E)	19	0.39	GGG(G)	31	0.52

Table S4. Uncorrected pairwise distance (P-distance) values among 21 species in the family Corydoradinae. Box borders indicate clades defined based on the phylogenetic tree.

Species	No.	1	2	3	4	5	6	7	8	9	10	11	12	13	14	15	16	17	18	19	20	21
H. sterbai	1							Clade 6														
H. julii	2	0.000																				
H. trilineatum	3	0.000	0.000																			
H. duplicareum	4	0.059	0.059	0.059																		
B. arcuatus	5	0.176	0.176	0.176	0.118																	
H. concolor	6	0.118	0.118	0.118	0.059	0.176																
H. panda	7	0.059	0.059	0.059	0.118	0.235	0.059															
O. aeneus	8	0.353	0.353	0.353	0.353	0.471	0.353	0.353			Clade 5											
O. rabauti	9	0.294	0.294	0.294	0.294	0.412	0.294	0.294	0.118													
H. cruziensis	10	0.353	0.353	0.353	0.412	0.529	0.412	0.353	0.294	0.235												
B. agassizii	11	0.353	0.353	0.353	0.412	0.529	0.353	0.294	0.353	0.412	0.412			Clade 4								
H. schwartzi	12	0.235	0.235	0.235	0.294	0.412	0.235	0.176	0.294	0.353	0.412	0.118										
B.multiradiatus	13	0.235	0.235	0.235	0.294	0.412	0.235	0.176	0.412	0.353	0.353	0.118	0.118									
H. nattereri	14	0.294	0.294	0.294	0.353	0.471	0.294	0.235	0.412	0.353	0.412	0.353	0.294	0.294		Clade 3						
H. paleatus	15	0.235	0.235	0.235	0.294	0.412	0.235	0.176	0.353	0.294	0.353	0.353	0.294	0.294	0.048							
S. barbatus-AE14	16	0.467	0.467	0.467	0.400	0.400	0.400	0.467	0.467	0.400	0.467	0.533	0.600	0.467	0.444	0.389				Clade 2		
S. barbatus-AR03	17	0.467	0.467	0.467	0.400	0.400	0.400	0.467	0.467	0.400	0.467	0.533	0.600	0.467	0.500	0.444	0.056					
S. barbatus-PE01	18	0.467	0.467	0.467	0.400	0.400	0.400	0.467	0.467	0.400	0.467	0.533	0.600	0.467	0.444	0.389	0.000	0.056				
S. barbatus-PS01	19	0.467	0.467	0.467	0.400	0.400	0.400	0.467	0.467	0.400	0.467	0.533	0.600	0.467	0.500	0.444	0.056	0.000	0.056			
G. hastatus	20	0.333	0.333	0.333	0.333	0.400	0.333	0.333	0.400	0.333	0.467	0.400	0.333	0.333	0.133	0.133	0.308	0.308	0.308	0.308		Clade 1
G. pygmeaus	21	0.533	0.533	0.533	0.533	0.533	0.467	0.467	0.467	0.533	0.667	0.400	0.400	0.400	0.600	0.533	0.400	0.400	0.400	0.400	0.615	

Reference
